# Safety and tolerability study of sotrovimab (VIR-7831) prophylaxis against COVID-19 infection in immunocompromised individuals with impaired SARS-CoV-2 humoral immunity

**DOI:** 10.1186/s40814-023-01325-y

**Published:** 2023-06-16

**Authors:** Isabel H. Gonzalez-Bocco, Katherine Beluch, Alyssa Cho, Chloe Lahoud, Fabiola A. Reyes, Dimitrios G. Moshovitis, Gillian M. Unger-Mochrie, Wei Wang, Sarah P. Hammond, Jennifer Manne-Goehler, Sophia Koo

**Affiliations:** 1 Division of Infectious Disease, Dana-Farber Cancer Institute, Boston, MA USA; 2 Division of Infectious Disease, Brigham and Women’s Hospital, Boston, MA USA; 3 Harvard Medical School, Boston, MA USA; 4 Medicine Department, Brigham and Women’s Hospital, Boston, MA USA; 5 Division of Infectious Disease, Massachusetts General Hospital, Boston, MA USA

**Keywords:** COVID-19, Immunocompromised Host, Pre-exposure prophylaxis, Antibodies, Monoclonal, Humanized, Immunity

## Abstract

**Background:**

Multiple vaccines have been approved since August 2021 to prevent infection with SARS-CoV-2; however, 20–40% of immunocompromised people fail to develop SARS-CoV-2 spike antibodies after COVID-19 vaccination and remain at high risk of infection and more severe illness than non-immunocompromised hosts. Sotrovimab (VIR-7831) is a monoclonal neutralizing antibody that binds a conserved epitope on the SARS-CoV-2 spike protein. It is neither renally excreted nor metabolized by P450 enzymes and therefore unlikely to interact with concomitant medications (e.g., immunosuppressive medications). In this open-label feasibility study protocol, we will define the optimal dose and dosing interval of sotrovimab as pre-exposure prophylaxis for immunocompromised individuals as well as its safety and tolerability in this population specifically.

**Methods:**

We will enroll 93 eligible immunocompromised adults with a negative or low-positive (< 50 U/mL) SARS-CoV-2 spike antibody. In phase 1, the first 10 patients will participate in a lead-in pharmacokinetics (PK) cohort study to determine the optimal dosing interval. Phase 2 will expand this population to 50 participants to examine rates of infusion-related reactions (IRR) with a 30-min 500 mg sotrovimab IV infusion. Phase 3 will be an expansion cohort for further assessment of the safety and tolerability of sotrovimab. In phase 4, the first 10 patients receiving 2000 mg IV of sotrovimab on the second sotrovimab infusion day will comprise a lead-in safety cohort that will inform the duration of observation following administration of the drug. The patients will be followed for safety and COVID-19 events for 36 weeks after the second dose.

**Discussion:**

In a previous phase III randomized, placebo-controlled pivotal trial, there were no significant differences in the prevalence of adverse events in patients receiving sotrovimab vs. placebo. Thus, we propose an open-label feasibility study protocol of sotrovimab as pre-exposure prophylaxis for immunocompromised individuals to evaluate its PK in immunocompromised individuals with impaired SARS-CoV-2 humoral immunity and define optimal dosing intervals. We also aim to determine COVID-19 infections over the study period and self-reported quality of life measures throughout the study.

**Trial registration:**

ClinicalTrials.gov Identifier: NCT05210101.

## Introduction

Severe acute respiratory syndrome coronavirus 2 (SARS-CoV-2) is a novel single-stranded RNA betacoronavirus first identified in December 2019 that has spread widely since [1–3]. The World Health Organization declared COVID-19 a global pandemic on March 11, 2020 [4]. And as of March 2022, there were over 6.1 million COVID-19-associated deaths worldwide [5].

The U.S. Food and Drug Administration (FDA) approved the Pfizer-BioNTech COVID-19 messenger RNA vaccine in August 2021, and in January 2022, the Moderna COVID-19 vaccine also received FDA approval [6]. Nevertheless, only 44–70% of patients exposed to anti-CD20 therapies, 73% of hematopoietic cell transplant recipients, and 14% of CD19-CAR-T cell recipients, for example, develop SARS-CoV-2 spike protein antibodies after two doses of SARS-CoV-2 mRNA vaccination [7–11]. Patients with B cell hematologic malignancies, such as chronic lymphocytic leukemia (37–64%), follicular lymphoma (78%), and diffuse large B cell lymphoma (79%), also have significantly impaired humoral responses to SARS-CoV-2 mRNA vaccination, especially patients treated with anti-CD20 antibodies, steroids, venetoclax, or Bruton’s tyrosine kinase inhibitors, as well as patients who have not recently received chemotherapeutic agents [12–17]. In two studies of solid organ transplant patients receiving immunosuppressive drugs, the prevalence of SARS-CoV-2 spike antibodies was 20–40% after two doses of the vaccine [18, 19]. While a third booster dose of mRNA vaccine increases immunogenicity of the SARS-CoV-2 mRNA vaccines, with a detectable serologic response rising from 40% after dose 2 to 68% following dose 3, a substantial subset of solid organ transplant patients still does not develop evidence of humoral immunity to SARS-CoV-2 [19–21]. Patients with rheumatologic diseases receiving anti-CD20 therapies and other immunodeficiency syndromes have similarly impaired humoral immune responses to SARS-CoV-2 vaccination [7, 8, 22–25]. Additionally, immunocompromised patients are also at higher risk of severe COVID-19 disease, highlighting the need for alternative approaches to prevent SARS-CoV-2 infection in these particularly vulnerable patients [26, 27].

Monoclonal antibodies are a highly target-specific and versatile alternative for COVID-19 prevention and treatment—they do not depend on the recipient’s immune response and are well-suited for immunocompromised patients with an impaired SARS-CoV-2 spike antibody response despite currently available vaccine products [28]. In May 2021, sotrovimab received FDA Emergency Use Authorization for the treatment of mild-to-moderate COVID-19 in adults and children [12 years of age and older weighing at least 40 kg] with positive results of direct SARS-CoV-2 viral testing at high risk of progression to severe COVID-19, including hospitalization and death [29, 30].

The primary aim of this study is to determine the feasibility of using sotrovimab for pre-exposure prophylaxis of SARS-CoV-2 infection and COVID-19 disease. Specifically, we will:

1. Evaluate PK data of sotrovimab in immunocompromised patients with impaired immunity against SARS-CoV-2 to determine optimal dose and dosing interval for future trials.

The secondary aim is to explore the impact of sotrovimab on patient-level to determine:

1. Safety and tolerability outcomes to guide the future selection of populations in further trials.
2. Proportion of study subjects who develop COVID-19 over the study follow-up period.
3. Quality of life measures over the study.

## Methods/design

### Study design

In this open-label feasibility study protocol, we will define the optimal dose and dosing interval of sotrovimab as pre-exposure prophylaxis for immunocompromised individuals as well as its safety and tolerability to then study the possibility of escalating this trial to determine efficacy. Sotrovimab will be administered in two sequential doses as prophylaxis in patients with impaired humoral immunity against SARS-CoV-2. A total of 93 patients will be enrolled in this study: 10 patients in a lead-in PK cohort to determine the optimal dosing interval between the first and second dose of sotrovimab and assess the safety and tolerability of the drug, 50 patients (including the 10 patients in the lead-in PK cohort) in a safety and tolerability lead-in cohort to examine rates of infusion-related reactions (IRR) with a 30-min 500 mg sotrovimab IV infusion, an expansion cohort of patients to further assess the safety and tolerability of sotrovimab in this patient population, with the sotrovimab infusion duration determined by the rate of IRRs in the 50-patient safety and tolerability lead-in cohort, and a 10 patient lead-in safety cohort for the second, 2000 mg dose of sotrovimab on treatment day 2—this cohort will inform the duration of observation following administration of the drug, with the observation period being reduced from 2 to 1 h for the remaining study subjects if none of these patients have a grade 3–4 infusion-related reaction with the 2000 mg dose.

### Inclusion criteria

Patients meeting the following inclusion criteria are eligible for study enrollment: (1) 18 years or older at the time of consent and weighing at least 40 kg; (2) having an immunocompromising condition that increases the likelihood of having an impaired humoral immune response to SARS-CoV-2 vaccination, such as exposure to an anti-CD20 monoclonal antibody for hematologic malignancy or an autoimmune/inflammatory disease in the 12-month period prior to consent, allogenic hematopoietic cell transplant ≥ 3 months and ≤ 1 year prior to consent, or allogeneic hematopoietic cell transplant > 1 year prior to consent plus active graft-versus-host disease on systemic immunosuppressive therapy, chimeric antigen receptor (CAR)-T cell therapy ≥ 4 weeks and ≤ 2 years prior to consent, chronic lymphocytic leukemia/small lymphocytic lymphoma (CLL/SLL), multiple myeloma, or Waldenström macroglobulinemia, solid organ transplant recipient receiving immunosuppressive therapy, congenital immunodeficiency syndrome, patients with hematologic malignancy or autoimmune/inflammatory disease exposed to immunosuppressive medications specifically associated with a blunted humoral immune response to SARS-CoV-2 vaccination (e.g., mycophenolate mofetil, azathioprine, methotrexate, Bruton tyrosine kinase inhibitors, ruxolitinib, venetoclax, or corticosteroids (prednisone > 20 mg or equivalent daily for at least 14 days) in the 3-month period prior to consent; (3) female participants either postmenopausal for at least 1 year, post-hysterectomy and/or post-bilateral oophorectomy, or in women of childbearing potential, having a negative urine or serum human chorionic gonadotropin pregnancy test prior to each sotrovimab dose and agreeing to use a highly effective method of birth control throughout the study period (e.g., oral, injected, transdermal, intravaginal or implanted hormonal form of contraception associated with inhibition of ovulation, placement of an intrauterine device or intrauterine hormone-releasing system, male partner sterilization, bilateral tubal occlusion, sexual abstinence); and (4) a negative or low-positive SARS-CoV2 spike antibody assay result within 28 days of consent (< 50 U/mL on a spike antibody assay available under FDA Emergency Use Authorization (EUA) such as the Roche Elecsys SARS-CoV-2 semiquantitative spike antibody assay).

### Exclusion criteria

Patients meeting the following exclusion criteria are ineligible for study enrollment: (1) active SARS-CoV-2 infection, with a positive SARS-CoV-2 RT-PCR or antigen test result within 21 days prior to consent; (2) symptoms suggestive of SARS-CoV-2 infection at screening; (3) close contact (less than 6 feet away for a cumulative total of ≥ 15 min over a 24-h period) with an individual with COVID-19 in the 14 days prior to consent; (4) individuals who are pregnant or breastfeeding; (5) receipt of any other investigational agents at the time of study enrollment; (6) individuals likely to have a life expectancy of less than 1 year, in the judgment of the investigator; (7) known hypersensitivity to any constituent present in sotrovimab or any other anti-SARS-CoV-2 monoclonal antibody products; (8) active enrollment on another interventional research study of any agent for the treatment or prophylaxis of SARS-CoV-2 infection; (9) exposure to any other anti-SARS-CoV-2 monoclonal antibody product for the treatment of COVID-19 in the prior 6 months; (10) exposure to any other anti-SARS-CoV-2 monoclonal antibody product for prophylaxis against COVID-19 infection in the prior 12 months; and (11) receipt of a SARS-CoV-2 vaccine dose within the 28 days prior to enrollment.

### Discontinuation criteria

Subjects may be discontinued from the study drug for the following reasons: (1) withdrawal of consent by the subject and/or the subject’s legally authorized representative; (2) loss to follow-up; (3) intercurrent illness that prevents further administration of study treatment; or (4) death.

In the following cases, the study drug will be withdrawn, but patients will continue to follow study procedures, such as (1) the occurrence of an AE that, in the investigator’s opinion, warrants discontinuation of the study drug, (2) pregnancy or the initiation of breastfeeding, (3) treatment with other monoclonal antibodies active against SARS-CoV-2 for postexposure prophylaxis after having a household contact with COVID-19 or for treatment of COVID-19 prior to treatment day 2. Participants removed from protocol therapy prior to dose 2 of the study drug for any of the reasons listed above will not receive a second dose of sotrovimab, but they will be asked to complete all other study assessments according to the study calendar.

### Recruitment

The planned sample size for this study will be 93 participants, all of whom will be accrued within 9–12 months, with follow-up visits for an additional 36 weeks after the last study subject receives their last sotrovimab dose, for a total study duration of approximately 2 years. A total cohort size of 93 subjects (inclusive of the 10 patients in the lead-in PK cohort) will provide a suitably precise assessment of the descriptive safety and tolerability profile for a two-dose regimen of IV sotrovimab. This enrollment plan is consistent with similar ongoing clinical studies of COVID-19 prophylaxis and justified by the relatively broad inclusion criteria for the study, encompassing multiple distinct subgroups of immunocompromised patients. Patients will be enrolled at Brigham and Women’s Hospital, Dana-Farber Cancer Institute, and Massachusetts General Hospital in Boston, MA, USA.

### Interventions

Pre-treatment procedures include confirmation that the potential study subject meets inclusion and exclusion criteria and that the participant has a negative or low-positive (< 50 U/mL) SARS-CoV-2 spike antibody test result within 28 days of consent using an assay available under FDA EUA. Potential study subjects who have not yet had their SARS-CoV-2 spike antibody testing performed in this window should be tested. If the study subject meets inclusion and exclusion criteria, screening and treatment day 1 procedures may be performed on the same day. Informed consent will be completed during the screening visit.

Study subjects will receive sotrovimab 500 mg as an intravenous (IV) infusion over 30 min on treatment day 1 and 2000 mg as an intravenous (IV) infusion over 60 min on treatment day 2. Study subjects will be monitored for 1 h after the end of the first infusion; for the second infusion, the first 10 study subjects will be monitored for 2 h (second lead-in safety cohort for the 2000 mg dose), with a 1-h monitoring period in all subsequent patients as long as there is no grade > 2 infusion-related reactions in this second lead-in safety cohort. Prior to the spread of the BA.2 variant, which made it necessary to administer a repeat sotrovimab dose earlier than originally anticipated, using theoretical modeling and logistical considerations, the interval between the two doses was initially going to be determined by modeling the PK data obtained from a 10-person lead-in PK cohort and is anticipated to be between 12 and 24 weeks in length, given the half-life of sotrovimab.

Following the schedule outlined in Table 1, physicians will collect the following:

- Vital signs including body temperature, heart rate, blood pressure, oxygen saturation (SpO2), and respiratory rate
- Karnofsky performance status
- An electrocardiogram (ECG)
- Pregnancy testing in women of childbearing potential. A negative urine or serum hCG test is required prior to each dose of sotrovimab
- Local laboratory testing at each study site, including hematology (complete blood count and differential) and blood chemistry (basic metabolic panel and liver function tests) assays at each time point outlined in Table 1
- Serum sotrovimab levels for PK assessment

**Table 1:**
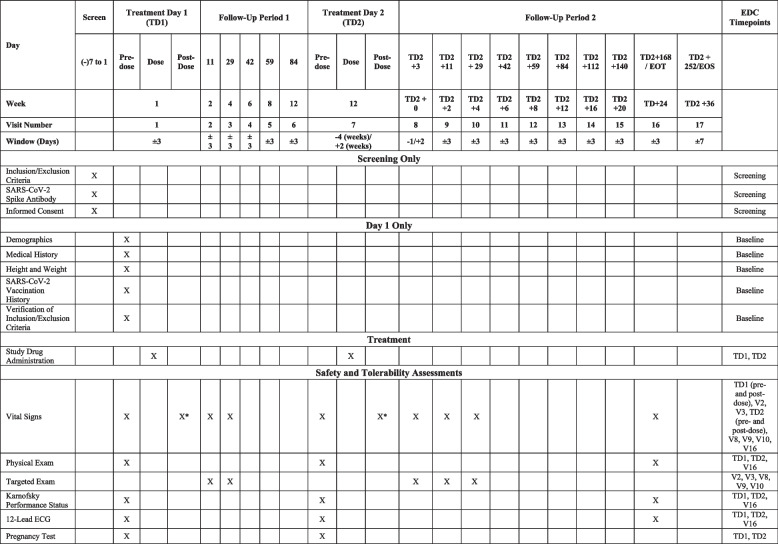
Study calendar

^a^Vital signs should be assessed within 30 minutes after the end of the infusion

Self-collected anterior nasal swabs will be obtained by study subjects biweekly over the study period using the binx Health At-Home Nasal Swab COVID-19 Sample Collection Kit, which has FDA EUA for the self-collection of anterior nasal swab specimens at home, for SARS-CoV-2 RT-PCR [31, 32].

COVID-19 symptom assessment will be performed at each in-person or remote study visit using the Center for Disease Control and Prevention’s (CDC) list of symptoms commonly associated with COVID-19 [33]. Study participants will be asked if they have any of these symptoms de novo or worsening from baseline at each in-person and remote visit, using a questionnaire. If they report any of these symptoms (new or worsening from baseline) at an in-person visit, they will have a nasopharyngeal swab obtained for local laboratory RT-PCR testing for SARS-CoV-2. If they report any of these symptoms at a remote visit, they will be asked to go to a local laboratory for SARS-CoV-2 RT-PCR testing.

A comprehensive physical exam will be performed according to the schedule outlined in Table 1. The targeted physical examination will include, but is not limited to, assessment of the following: vital signs, the general appearance of the study subject, lung exam (e.g., crackles, rhonchi, diminished breath sounds), and skin exam (e.g., urticaria, ulcerations, erythema multiforme, maculopapular rashes).

Patients diagnosed with COVID-19 during the follow-up period will be assessed using the 8-point National Institute of Allergy and Infectious Diseases ordinal scale (NIAID-OS) at the end of hospitalization or those who do not require hospitalization, 14 days after the diagnosis of COVID-19.

Health-related quality of life measures will be assessed using the RAND 36-Item Short Form Health Survey (SF-36) instrument [34]. Study subjects will complete these surveys at certain study visits, as outlined in Table 1.

### Participant timeline

The screening window prior to study entry will last up to 7 days (day − 7 to treatment day 1). The study period will last from treatment day 1 to the end of the treatment visit, and patients will be followed according to the schedule of in-person and remote study visits, assessments, and sample collection points as outlined in Table 1. One hundred sixty-eight days after treatment day 2, patients will have an in-person end of treatment (EOT) visit for sample collection and assessments. A final remote end-of-study (EOS) visit will be conducted over the phone 36 weeks/252 days after each subject’s final dose of sotrovimab. Participants will be followed for 36 weeks in total after their second sotrovimab dose, corresponding approximately to 5 half-lives of sotrovimab since the final dose, or death, whichever occurs first. Participants removed from protocol therapy due to unacceptable adverse events (AEs) will be followed until the resolution or stabilization of these AEs.

### Outcome measures

#### Primary outcome measures

##### Sotrovimab pharmacokinetics

We will evaluate the pharmacokinetics (PK) of sotrovimab in serum samples collected from study subjects to define the optimal dose and dosing interval for further trials.

#### Secondary outcome measures

##### Sotrovimab safety and tolerability

We will determine the proportion of study subjects with treatment-emergent grade 3–4 AEs, as defined by DAIDS grading criteria, the proportion of study subjects with treatment-emergent serious adverse events (SAEs), and the proportion of study subjects with AESI, including infusion-related and hypersensitivity reactions, the development of anti-drug antibody (ADA) levels, and antibody-dependent enhancement (ADE) of COVID-19 disease.

##### COVID-19 infection

We will measure the proportion of study subjects who develop COVID-19 (of any severity) over the study follow-up period and the proportion of study subjects with severe COVID-19, with any of the following criteria: (a) dyspnea, respiratory rate of 30 or more breaths per minute, blood oxygen saturation of 93% or less, a ratio of the partial pressure of arterial oxygen to the fraction of inspired oxygen (PaO_2_:FiO_2_) < 300 mm Hg, or infiltrates in more than 50% of the lung field; (b) emergency department (ED) visit, inpatient hospitalization, or intensive care unit (ICU) hospitalization within 28 days of a new diagnosis of SARS-CoV-2; (c) need for new or increasing supplemental oxygen or mechanical ventilation within 28 days of a new diagnosis of SARS-CoV-2; or (d death due to any cause during the study follow-up period. In patients who develop COVID-19, we will assess the greatest extent of COVID-19 symptoms, according to the 8-point National Institute of Allergy and Infectious Diseases ordinal scale (NIAID-OS), at the end of hospitalization or 14 days after the diagnosis of COVID-19 [33].

##### Quality of life

We will assess health-related quality of life measures over the study using the Short Form Health Survey (SF-36) instrument, assessed at baseline, 12 weeks, and 24 weeks [35].

Feasibility criteria for pharmacokinetics will need to match prior popPK analysis from prior clinical studies in immunocompetent non-hospitalized patients [36], expected to provide at least 3 months of protection for immunocompromised hosts. For patient-centered data, no major SAEs associated with the study drug. Each of the outcome measures would help guide modifications required to the study design and intervention for further large-scale trials.

### Safety and adverse effects

Adverse events of special interest (AESI) will be evaluated from patient consent until the end of the study visit 36 weeks after treatment day 2. AESI is defined as relevant known toxicities of therapeutic monoclonal antibodies that the study team will monitor during the study, including hypersensitivity reactions or anaphylaxis, infusion-related reactions, and immunogenicity-related AEs (development of ADA).

All grade 3–4 TEAEs and all treatment-emergent SAEs, defined using DAIDS grading criteria, will be collected from patient consent until the end of the study remote visit, 36 weeks after treatment day 2 [37]. Patients will be solicited for the development of any AE at each in-person or remote visit, and any unsolicited AEs reported will be investigated further.

### Sample size

The planned sample size for this study is 93 participants, all of whom will be accrued within 9–12 months, with follow-up visits for an additional 36 weeks after the last study subject receives their last sotrovimab dose, for a total study duration of approximately 2 years. A total cohort size of 93 subjects, including the 10 patients in the lead-in PK cohort and in the 2000 mg dose lead-in cohort, will provide a suitably precise assessment of the descriptive safety and tolerability profile for a two-dose regimen of IV sotrovimab. This enrollment plan is consistent with similar ongoing clinical studies of COVID-19 prophylaxis and justified by the relatively broad inclusion criteria for the study, encompassing multiple distinct subgroups of immunocompromised patients. Table 2 contains estimates of precision in the estimated grade 3–4 TEAE rate.

**Table 2:** Precision in the estimated grade 3–4 treatment-emergent adverse event rate with a total cohort size of 93 patients

AE rate	Exact 95% confidence interval	Probability of seeing at least one event
**1%**	(0.0%, 5.8%)	0.607
**2%**	(0.7%, 9.1%)	0.847
**3%**	(1.2%, 10.6%)	0.941
**4%**	(1.2%, 10.6%)	0.978
**5%**	(1.8%, 12.1%)	0.992

Patients who discontinue their participation in the study prematurely will not be replaced. The study will end on the date when the last living patient completes the last study visit, withdraws from the study, or is lost to follow-up. The accrual targets by study subject race and ethnicity are outlined in Table 3.

**Table 3:** Study accrual targets by race/ethnicity

Accrual targets					
	**Sex/gender**				
	**Females**		**Males**		**Total**
**Ethnic category**					
Hispanic or Latino	3	+	3	=	6
Not Hispanic or Latino	43	+	44	=	87
**Ethnic category: total of all subjects**	46	+	47	=	93
**Racial category**					
American Indian or Alaskan Native	0	+	0	=	0
Asian	2	+	2	=	4
Black or African American	4	+	4	=	8
Native Hawaiian or other Pacific Islander	0	+	0	=	0
White	40	+	41	=	81
**Racial category: total of all subjects**	46	+	47	=	93
	(A1 = A2)		(B1 = B2)		(C1 = C2)

### Data management and monitoring

An analysis of the 10-patient PK lead-in cohort and theoretical PK modeling of the duration of the antiviral efficacy of sotrovimab based on the rising prevalence of the SARS-CoV-2 Omicron BA.2 variant will define the optimal dosing interval between two sequential doses of intravenous sotrovimab. The remaining participants will follow the dosing interval defined by this lead-in cohort. The 50-patient safety and tolerability lead-in cohort (inclusive of these 10 study participants) will inform the infusion period for the remaining 43 participants. If there are no grade ≥ 2 infusion-related reactions in this initial group, patients in the expansion cohort will receive their intravenous sotrovimab doses over a 15-min infusion period; however, if there is at least one patient with a grade ≥ 2 infusion-related reaction, the study subjects in the expansion cohort will continue to receive intravenous sotrovimab over a 30-min infusion period. Finally, the 10 patients in the 2000 mg dose sotrovimab lead-in cohort will inform the observation period for all patients who receive their 2000 mg dose after these patients, with a 2-h observation period following completion of the 2000 mg dose for these 10 patients, reduced to a 1 h period for all subsequent patients if there are no grade > 2 infusion-related reactions or SAEs potentially related to the study drug in the lead-in safety cohort. These data will be reviewed by the study team.

Syneos Health will provide electronic data capture services and monitor and perform quality checks on data collected from this study. Investigative sites are responsible for submitting data and/or data forms into the electronic case report forms designed to capture specific and relevant study data.

### Statistical analysis

No formal hypothesis tests will be performed, and no formal significance tests for comparisons will be made. Descriptive statistics will be provided for selected PK parameters of interest for the cohort. All PK parameters will be summarized in terms of means, standard deviation (SD), % coefficient of variation (CV), range, median, and the number of samples. Clinical expertise will inform the interpretation of these safety and tolerability data and PK values.

The secondary endpoints of interest in this study will include the following: (1) safety and tolerability outcomes of interest will be displayed in the form of listings, frequencies, summary statistics, and graphs, where appropriate. Binary data will be presented in the form of counts and proportions, and continuous data will be presented using descriptive statistics (*N*, mean, SD, median, and range). (2) COVID-19 infections over the study period and (3) self-reported quality of life measures throughout the study. COVID-19 infections in this cohort will be summarized using standard descriptive statistics, including counts and proportions where applicable. Logistic regression models will be used to examine sociodemographic and health-related factors associated with incident COVID-19 infection in this population over the study period. Covariates such as age, sex, type of immunocompromising condition, prior vaccination status, and body mass index will be assessed in these models. Changes in self-reported quality of life from baseline to the study mid-point and baseline to end of treatment, as assessed by the SF-36 instrument, will be analyzed descriptively and, where appropriate, graphically visualized from baseline to end of treatment. A linear mixed model with a random slope for each participant and an unstructured correlation matrix will be used to examine changes in SF-36 continuous scores over time and include baseline demographic and relevant clinical parameters as fixed effects.

Where possible, all analyses will be conducted in the full cohort and separately, stratified by subtype of underlying immunocompromised status as follows: (1) solid organ transplant recipients, (2) participants with a history of hematologic malignancy requiring hematopoietic cell transplant and/or history of chimeric antigen receptor (CAR)-T cell therapy, and (3) participants with any other hematologic malignancy, autoimmune, or inflammatory condition requiring either the use of an anti-CD20 monoclonal antibody or other immunosuppressive medication specifically associated with a blunted humoral immune response to SARS-CoV-2, as outlined in the inclusion criteria. These analyses, including comparisons of the primary and secondary outcomes of interest across these three groups, will permit an exploration of specific differences that may be present between subgroups with varying immunological deficits.

### Quality control

In accordance with the International Council for Harmonization of Technical Requirements for Pharmaceuticals for Human Use (ICH) E6, the sponsor-investigator is responsible for quality control and quality assurance that the study is conducted and the data are generated, recorded, and reported in compliance with the protocol, good clinical practice (GCP), and any applicable regulatory requirements. All research personnel participated in the study initiation visit, including protocol and quality control assurance training.

Investigators at each site must allow study-related monitoring and provide access to all necessary facilities, study data, and documents for internal or external inspection or audit as needed; communicate any information arising from these inspections to the sponsor-investigator immediately; and take all appropriate measures requested by the sponsor-investigator and/or designees of the auditor to resolve any problems found during the audit or inspection.

## Discussion

This study evaluates PK data to define an adequate dosing interval and different dosages of sotrovimab as pre-exposure prophylaxis for individuals with impaired SARS-CoV-2 humoral immunity. Sotrovimab is an investigational engineered monoclonal antibody that targets a highly conserved epitope on the spike glycoprotein of SARS-CoV-2, for pre-exposure prophylaxis against COVID-19. This antibody has been modified in its Fc region to increase its binding affinity to the FcRn receptor in lysosomes in endothelial cells, rescuing these antibodies from degradation, prolonging its serum half-life, and potentially increasing its bioavailability in tissues such as the respiratory mucosa. Sotrovimab targets a highly conserved spike epitope, with amino acid conservation > 99.99% for all amino acids based on > 2,100,00 available sequences. This study will assess two IV doses of sotrovimab, administered on treatment day 1 and on a second dosing day approximately 8–14 weeks after the first dose. The dosing interval will be determined based on modeling of the duration of efficacy of sotrovimab as antiviral prophylaxis against the Omicron BA.2 subvariant [34, 38, 39].

The primary medical approach to preventing COVID-19 is currently centered on several widely available vaccine products. While vaccine products offer an excellent approach to the prevention of COVID-19 disease, emerging evidence has demonstrated that a high proportion of immunocompromised patients, including patients exposed to anti-CD20 therapies, hematopoietic cell transplant recipients, CD19-CAR-T cell recipients, and solid organ transplant recipients, do not develop evidence of humoral immunity to SARS-CoV-2 [19–21]. Therefore, alternative preventive approaches are needed in these patients, such as antiviral and monoclonal antibody therapies.

Sotrovimab retains activity against alpha (B.1.1.7), beta (B.1.351), gamma (P.1), delta (B.1.617.2), kappa (B.1.617.1), and omicron (BA.1) variant live virus; against omicron (BA.2 and BA.3), there are fold changes of 15.7 and 7.3, respectively, in a pseudotyped virus system, and 35.1 against BA.2 live virus. In March 2022, the distribution of sotrovimab for the treatment of mild to moderate SARS-CoV-2 infection was paused in regions where the Omicron BA.2 variant constitutes more than 50% of circulating variants, given the reduced antiviral susceptibility of the drug against it, then the EUA ultimately withdrawn as the BA.2 variant became the dominant circulating variant in the USA [40–43]. However, the FDA allowed this trial to proceed despite the advent of the Omicron BA.2 variant, given the planned increase of the sotrovimab dose to 2000 mg for the second drug administration at 8–14 weeks after the first dose and the use of this agent for prophylaxis in immunocompromised patients in this study.

The exposure after 2000 mg IV was predicted using a preliminary population PK model that was developed using data across several studies, including COMET-ICE, COMETPEAK, BLAZE-4, and a PK study in individuals of Japanese and Caucasian descent. The predicted median (10th, 90th percentile) sotrovimab serum concentrations at 16 weeks following a second sotrovimab dose of 2000 mg IV given 8 or 12 weeks after the first 500 mg IV dose are 69.2 (38.7, 109.4) μg/mL and 64.5 (38.0,105.2) μg/mL, respectively. Tissue-adjusted EC90 values were calculated using different assumptions for lung to serum ratio of 25%, 15%, or 10% (based on literature reports for monoclonal antibodies in general and were used to estimate coverage above tissue-adjusted EC90 following the second dose of sotrovimab 2000 mg IV). When assuming a lung to serum ratio of 25%, a 2000 mg IV dose is expected to provide coverage against the Omicron BA.2 variant in 90% of patients for approximately 4 months when given 8–12 weeks after the first dose. With more conservative assumptions of a lung to serum ratio of 15% and 10%, a 2000 mg IV dose given 8–12 weeks after the initial 500 mg IV dose is expected to provide adequate protection against BA.2 in 90% of patients for approximately 2 months [44–49].

Sotrovimab is also neither renally excreted nor metabolized by cytochrome P450 (CYP) enzymes; therefore, interactions with concomitant medications that are renally excreted or that are substrates, inducers, or inhibitors of CYP enzymes are unlikely, making it well-suited for use in immunocompromised patients, who tend to receive multiple concomitant treatments with potential for drug-drug interactions. In vitro pharmacodynamic studies with remdesivir or bamlanivimab and sotrovimab showed additive virologic effects and no antagonism [29].

This study aims to evaluate PK data of sotrovimab in immunocompromised individuals with impaired humoral immunity to define the optimal dose and dosing interval as a pre-exposure prophylaxis drug against SARS-CoV-2 infection and COVID-19 disease in this population. Yet, it is not powered to assess efficacy against SARS-CoV-2 infection initially because there is no placebo arm; therefore, further studies should aim to elucidate this. Additionally, exploring the impact of sotrovimab on patient-level safety and tolerability outcomes will guide the future selection of populations in further trials.

### Trial status

This trial is operating under the original protocol version dated January 4, 2022. Recruitment began on 7 February 2022. Recruitment ended on April 1, 2022, and we anticipate that our last patient’s last visit date will be around March 2023.

## Data Availability

The sponsor-investigator and study investigators will have access to the final trial dataset. The sponsor is not allowed to share access to the trial data with any commercial organization other than GSK or VIR (or GSK’s nominees) for a period of 5 years from the date of study conclusion without the prior written consent of GSK and VIR.
